# Study of predictive factors for response to ^177^LU-PSMA in patients with metastatic castration-resistant prostate cancer

**DOI:** 10.3389/fmed.2025.1538507

**Published:** 2025-03-17

**Authors:** Hugo Peslier, Valérie Seegers, Pierre-Alban Dufour

**Affiliations:** ^1^Department of Nuclear Medicine, Institut de Cancérologie de l’Ouest (ICO), Angers, France; ^2^Research and Statistics Department, Institut de Cancérologie de l’Ouest (ICO), Angers, France

**Keywords:** ^177^Lu-PSMA, mCRPC, predictive factors, therapeutic response, SULmax, total tumor volume, PSG score

## Abstract

**Introduction:**

Metastatic castration-resistant prostate cancer (mCRPC) is an aggressive disease with a poor prognosis and few therapeutic options. In recent years, ^177^Lu-PSMA, a novel radioligand therapy, has shown promising results in patients who have failed conventional therapies. However, around 30% of patients do not respond adequately to this treatment. In this retrospective cohort study, we examined clinical, biological, and ^68^Ga-PSMA PET/CT-derived factors associated with poor treatment response.

**Materials and methods:**

We conducted a retrospective cohort study including 63 patients treated at ICO Angers for progressive mCRPC following Novel Hormonal Agents and taxane-based chemotherapy. The primary endpoint was early treatment discontinuation, defined as stopping therapy at or before the 4th cycle. Secondary endpoints included PSA response and overall survival.

**Results:**

A total of 63 patients were included in the study. Factors associated with early treatment discontinuation included a BMI < 25 kg/m^2^, PSA doubling time < 2 months, hemoglobin levels <10 g/dL, albumin levels <35 g/L, lactate dehydrogenase (LDH) levels >250 IU/L and alkaline phosphatase (ALP) levels >125 IU/L. On ^68^Ga-PSMA PET/CT imaging, low SUL_max_, high Total Tumor Volume, and a low PSG score were also linked to early treatment discontinuation.

**Conclusion:**

This study identified several clinical, biological, and ^68^Ga-PSMA PET/CT-derived factors associated with early treatment discontinuation. Patients with poor overall health, aggressive or extensive disease, or low PSMA expression are at higher risk of treatment failure.

## Introduction

According to the Global Cancer Observatory, prostate cancer is the second most common cancer and the fifth leading cause of cancer-related deaths in men worldwide in 2020, with an incidence rate of 30.7 per 100,000 and a mortality rate of 7.7 per 100,000 (1).

In France, according to 2018 data from INCA (Institut National du Cancer), prostate cancer is the most common cancer and the third leading cause of cancer-related deaths in men. The median age at diagnosis is 69 years. It is a condition with a favorable prognosis, with a 5-year survival rate of 93%. Indeed, 80% of cases are diagnosed at a localized stage, and the stage of the disease at diagnosis is a major prognostic factor (2). Notably, the 5-year survival rate of *de novo* metastatic prostate cancer is around 30% (3).

In the natural evolution of untreated prostate cancer, initial localized disease slowly progresses to locoregional involvement, and subsequently to a metastatic stage with bone, lymph node or visceral metastases. Following definitive local therapy for localized forms through radical prostatectomy or external beam radiotherapy, recurrence is observed in 27–53% of patients (4).

At the metastatic castration-resistant stage defined by biochemical or radiological disease progression in an environment with very low serum testosterone concentration (< 50 ng/dL), prostate cancer is characterized by a poor prognosis and the available therapeutic options are currently limited to anti-hormonal deprivation therapy (ADT) in combination with novel hormonal agents (NHA) (e.g., enzalutamide, abiraterone) or taxane-based chemotherapies (e.g., docetaxel and cabazitaxel). For patients with metastatic castration-resistant prostate cancer (mCRPC) with alteration in BRCA 1 or 2, Olaparib can be considered after NHA (5).

For several decades, extensive research has sought to identify cellular targets for the development of novel targeted therapies in prostate cancer. In 1987, Horoszewicz et al. first described Prostate-Specific Membrane Antigen (PSMA) (6). This is a type II transmembrane protein involved in tumor proliferation and neoangiogenesis through the metabolism of glutamate and folates. PSMA is overexpressed in most prostate cancers, approximately 1,000 times more than in normal prostatic tissue, but is also physiologically expressed in various other tissues, particularly the salivary glands. Importantly, PSMA is not exclusive to prostate cancer; it is also present on endothelial cells of neovessels in other tumors, including colon adenocarcinoma, renal cell carcinoma, lung cancer, and melanoma. Studies have shown that PSMA expression correlates with tumor grade and the presence of metastatic disease, with higher levels associated with poorer prognosis.

In the field of nuclear medicine, several synthetic ligands of PSMA have been developed with the aim of creating radiopharmaceutical drugs both for imaging and therapy (7, 8). This refers to the concept of theranostics, a term derived from the combination of ‘therapy’ and ‘diagnostics.’ Theranostics involves using the same specific ligand for a cellular target, initially labeled with an isotope dedicated to imaging (e.g., ^99m^Tc for SPECT imaging, or ^68^Ga and ^18^F for PET/CT imaging). In a subsequent step, the ligand is combined with an alpha or beta- emitting radioisotope capable of destroying the previously targeted tumor cells (for exemple ^177^Lu wich is a beta- emitter, or ^225^Ac wich is an alpha emitter) (9).

Among the radioisotopes suitable for therapy, ^177^Lu is a beta-emitting radionuclide with a half-life of 6.65 days, a maximum *β*- particle energy of 497 keV and a maximum tissue penetration of 1.8 mm. This makes it particularly suitable for targeted treatment of prostate cancer tumors and their microenvironment, minimizing damage to surrounding healthy tissue. Gamma emission with 113 and 208 keV photons allows for whole-body scintigraphy after each treatment cycle to verify the proper uptake of the radiopharmaceutical drug to all tumor targets, ensure the absence of extravasation and to perform personalized dosimetry. Comparison of each post-therapy scintigraphy also allows tracking treatment efficacy and detecting any potential disease progression that would necessitate modification of the therapeutic approach (10, 11).

The international open-labeled phase 3 trial VISION involving 831 patients, investigating the efficacy and safety of ^177^Lu-PSMA-617 (commercialized under the name PLUVICTO^®^), has shown significant promise in extending imaging-based progression-free survival (median: 8.7 vs. 3.4 months) and overall survival (median: 15.3 vs. 11.3 months) when administered alongside standard-care therapy in patients with advanced and progressive PSMA-positive mCRPC, ^177^Lu-PSMA-617 was associated with a low incidence of serious adverse events. In this study, patients were required, among other criteria, to have previously received one NHA, at least one but no more than two taxane regimens, a positive ^68^Ga-PSMA-11 PET-CET scan, an Eastern Cooperative Oncology Group (ECOG) Performance Status score of 0 through 2, a life expectancy >6 months and an adequate organ function. The positivity criteria for ^68^Ga-PSMA PET/CT in the VISION study are presented below in Table 1 (12). Based on PSMA PET/CT criteria, patients who were classified as screen failures in the VISION trial had worse short-term outcomes than those who were classified as eligible (13).

**Table 1:** Interpretation criteria for ^68^Ga-PSMA PET in the VISION study.

Positive ^68^Ga-PSMA PET/CT	Négative ^68^Ga-PSMA PET/CT
At least one PSMA-positive lesion *And* No PSMA-negative lesion	No PSMA-positive lesion *Or* Presence of at least one PSMA-negative lesion meeting the following size criteria: a short axis ≥ 2.5 cm for lymph nodes a short axis ≥ 1 cm for visceral or bone metastatic lesions
PSMA-positive lesion	PSMA-negative lesion
Lesion with uptake higher than that of the liver	Lesion with uptake equal or less than that of the liver

In France, PLUVICTO^®^ was first used in 2021 under a Temporary Authorization for Use. Since May 2023, it has been granted Marketing Authorization and is now routinely available for the treatment of adult patients with mCRPC who have progressive disease and are positive for PSMA, and who have been treated with androgen pathway inhibitor hormone therapy and taxane-based chemotherapy (14).

The initiation of treatment with PLUVICTO^®^ is complex and requires the expertise of a nuclear medicine physician in collaboration with an oncologist. Joint recommendations have been issued by the European Association of Nuclear Medicine (EANM) and the Society of Nuclear Medicine and Molecular Imaging (SNMMI) (15).

Before starting treatment, several eligibility criteria must be confirmed. In a theranostic approach, a PET scan with ^68^Ga-PSMA is required to identify PSMA-positive tumor targets and ensure there are no PSMA-negative lesions meeting the specified size criteria. Additionally, a standard biological assessment including a complete blood count, liver function tests and renal function tests must be conducted to rule out contraindications such as cytopenias or severe renal or hepatic insufficiency. A thorough evaluation of the patient’s general health and comorbidities should be performed on a case-by-case basis during a pre-treatment consultation. Relative contraindications established by nuclear medicine societies include: a life expectancy of less than 3 months, an ECOG Performance Status of ≥3, uncontrollable urinary incontinence, acute urinary obstruction, severe comorbidities (e.g., psychiatric or cardiovascular conditions affecting hydration), severe renal or hepatic insufficiency (GFR < 30 mL/min, creatinine > twice the upper limit of normal, liver enzymes > five times the upper limit of normal), active infection, and significant cytopenias (WBC < 2.5 G/L, ANC < 1.5 G/L PLT < 75 G/L).

The standard treatment regimen consists of 6 cycles administered every 6 weeks, each involving an intravenous injection of 7.4 GBq of ^177^Lu-PSMA-617. Due to the co-emission of a 208 keV gamma ray by ^177^Lu, a whole-body scan is performed within 48 h after each injection to confirm proper radiopharmaceutical binding to tumor targets, check for tumor progression that might impact treatment continuation and ensure there is no extravasation at the injection site or contamination.

During and after PLUVICTO^®^ administration, radioprotection measures must be implemented to prevent radioactive waste from contaminating the environment and to protect both healthcare staff and the patient’s close contacts. Specifically, patients should be hospitalized for 6 to 24 h to collect their urine in decay containers. Radioprotection guidelines for home care can be adjusted based on dose rate measurements taken at the end of hospitalization.

**Figure 1:**
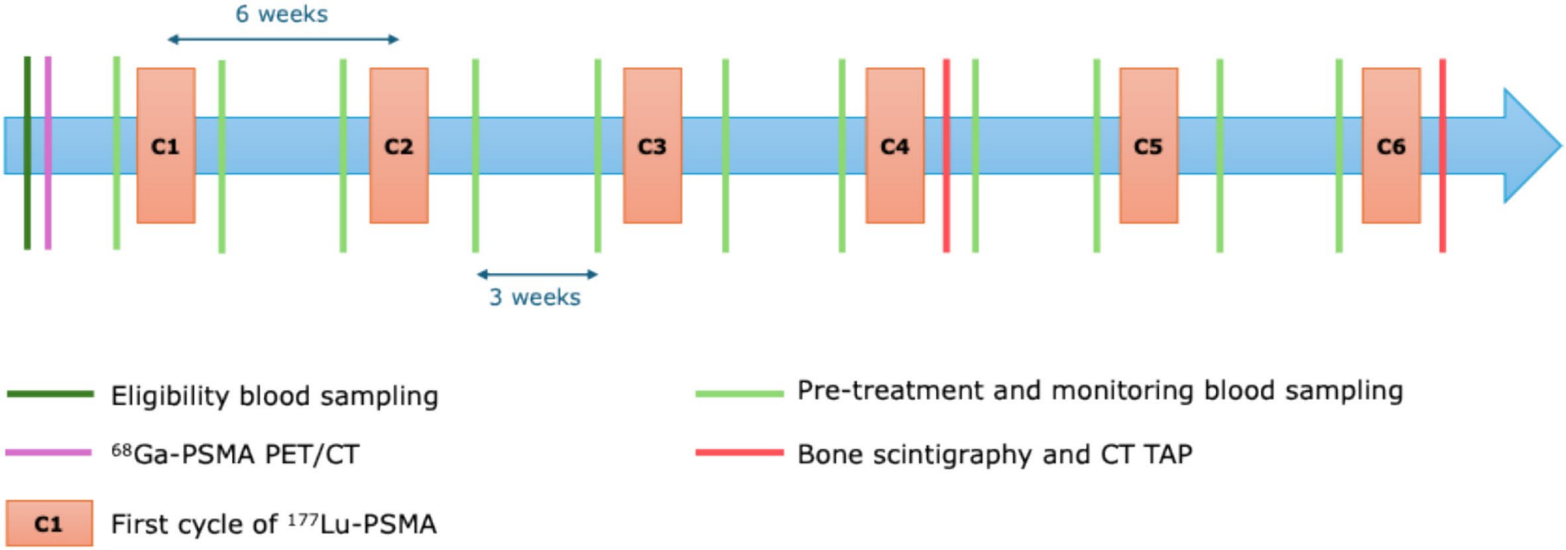
^177^Lu-PSMA treatment regimen and monitoring at ICO Angers.

Ongoing clinical and biological monitoring is essential during treatment, including assessing general health, screening for hematological and non-hematological toxicities and tracking PSA levels with tests conducted every 3 weeks. In cases of severe toxicity, adjustments to the dose, delays or discontinuation of treatment may be considered on an individual basis. Efficacy should be evaluated with Computed Tomography (CT) scans and bone scintigraphy every 12 weeks and at the end of the 6 treatment cycles. Indeed, as per the 2024 EAU – EANM – ESTRO – ESUR – SIOG Guidelines on Prostate Cancer, ^68^Ga-PSMA PET/CT is not yet recommended as imaging modality for therapeutic assessment in clinical practice. The treatment regimen for ^177^Lu-PSMA administered at ICO Angers is presented below in Figure 1.

Despite initial patient selection based on VISION study criteria, several retrospective studies indicate that approximately 30% of patients treated with ^177^Lu-PSMA-617 do not respond favorably (PSA decline <50% during treatment), and there is limited knowledge regarding predictive factors for treatment success (16).

To prevent administering a complex, costly, and potentially side-effect-laden treatment to patients who are unlikely to benefit, it is crucial to identify the patient groups most likely to respond to ^177^Lu-PSMA therapy. The aim of this study was to pinpoint clinical, biological and nuclear-imaging parameters in the pretherapeutic phase that are associated with a poor response to treatment.

## Methods

### Study design and population

We conducted a single-center, retrospective cohort including all consecutive patients treated at ICO Angers for mCRPC who received at least one cycle of ^177^Lu-PSMA between February 2022 and December 2023.

Inclusion criteria encompassed the presence of mCRPC, progression following at least one line of taxane chemotherapy and second-generation hormone therapy, and a positive ^68^Ga-PSMA PET/CT scan according to the VISION study criteria mentioned above.

Exclusion criteria included severe cytopenias and severe renal or hepatic insufficiency, as outlined in the PLUVICTO^®^ product’s Summary of Product Characteristics. Additionally, patients involved in clinical research protocols receiving experimental treatments during the ^177^Lu-PSMA therapy period, and those who did not consent to the use of their health data for research purposes, were also excluded.

The following data were collected from the patient’s electronic medical records: characteristics related to the initial disease and previously received treatments, clinical and biological characteristics at the start of ^177^Lu-PSMA treatment, and characteristics of the ^68^Ga-PSMA PET/CT scan before the first treatment cycle (C1).

Each treatment decision was made during a multidisciplinary tumor board meeting including oncologists and nuclear medicine physicians. During a pre-therapeutic consultation, patients were informed about the treatment modalities, expected benefits, and potential adverse effects. Written consent was systematically obtained. A consent form for the use of health data for research purposes was provided and completed by each patient who started treatment during the inclusion period. The study protocol was previously approved by an independent ethics committee.

### ^68^Ga-PSMA PET/CT and pre-treatment preparation

^68^Ga-PSMA PET/CT scans were performed in accordance with the 2017 European Association of Nuclear Medicine and Society of Nuclear Medicine and Molecular Imaging (EANM and SNMMI) standard recommendations. The first 15 patients underwent their scans at the ICO site in Nantes: 7 on a 2019 Siemens Biograph Vision PET scanner and 8 on a 2020 Siemens Biograph Vision PET scanner. 1 patient had his scan at the Institut Gustave Roussy in Paris on a Siemens Biograph Vision 600 PET scanner, and 47 patients underwent their scans at ICO Angers: 24 on a Philips VEREOS PET scanner and 23 on a GE Discovery IQ PET scanner.

Each scan was interpreted by an experienced nuclear medicine physician on a Syngo.via platform (Siemens Healthineers, Erlangen, Germany), including both visual and quantitative analyses. Lesions were considered PSMA-positive if their uptake intensity was equal to or greater than that of healthy liver tissue.

Measurements of Maximum Standardized Uptake Value – Lean body mass (SUL_max_) and Total Tumor Volume (TTV) were performed using the automatic segmentation module of the Syngo.via software, which allowed for automatic segmentation of all PSMA-positive lesions with a segmentation threshold of 41% of each lesion’s SULmax (this threshold is the commonly used in the literature for lesion segmentation on PET images). To quantify the uptake of tumor lesions, we chose to use SUL_max_, a semi-quantitative index that accounts for lean body mass, as opposed to SUV_max_ which is based on the total body weight. Indeed, due to significant differences in body morphotypes within the study population, the use of SUL_max_ allows for better interindividual comparability.

Patients were categorized into three groups based on the tracer uptake pattern for the definition of the PSG (PET Tumor-to-Salivary Gland) score, based on a visual analysis of the Maximum Intensity Projection image: patients whose more than 80% of lesions had higher uptake than the salivary glands were classified in the ‘High’ group; patients whose more than 80% of lesions had lower uptake than the salivary glands were classified in the ‘Low’ group; the remaining patients who did not fit into the first two categories were classified in the ‘Intermediate’ group. In an international multicenter retrospective study including 237 men, Hotta et al. demonstrated that this new visual score is predictive of the response to ^177^Lu-PSMA treatment with better PSA reduction and longer overall survival for ‘High’ scores, and with a substantial interreader reproducibility (17).

### ^177^Lu-PSMA therapy protocol

The ^177^Lu-PSMA treatment protocol consisted of administering 6 cycles of 7,4 GBq, each spaced 6 weeks apart, in the context of day hospitalization. At the end of each cycle, whole-body scintigraphy was performed to verify the proper distribution of the radiopharmaceutical to the tumor targets, the absence of extravasation at the injection site or contamination, and the absence of new uptake sites that could suggest disease progression during treatment.

Regular clinical and biological monitoring was established, with laboratory tests performed within 7 days before each cycle and 3 weeks after each treatment cycle. These tests included PSA level measurement to track changes, complete blood counts, comprehensive liver and kidney function tests, and albumin levels to ensure the treatment’s tolerability. Each patient was regularly consulted by both their treating oncologist and the nuclear medicine physician overseeing the treatment. In cases of severe adverse effects, such as cytopenias detected during monitoring, decisions regarding the spacing, postponement or permanent discontinuation of treatment cycles were made on a case-by-case basis by the multidisciplinary medical team.

At the end of the fourth cycle (C4) and the sixth cycle (C6), bone scintigraphy and CT scans of the thorax, abdomen, and pelvis (TAP) were performed to evaluate therapeutic efficacy. An earlier interim evaluation could be requested in case of suspected disease progression during treatment, before C4 or C6.

### Outcomes

The primary endpoint was poor response to ^177^Lu-PSMA, defined by early discontinuation of treatment between the first dose and post-C4 therapeutic assessment for any reason (e.g., progression objectively confirmed by CT or bone scan, or early treatment interruption due to severe hematological toxicity or deterioration in general condition, where continuing treatment was considered unreasonable). Secondary endpoints included a lack of treatment response as measured by PSA levels and the description of overall survival in treated patients. The lack of response based on PSA levels was defined as the proportion of patients who did not achieve a reduction of more than 50% from their baseline PSA level, as measured on the last biological assessment performed prior to C1.

### Statistical analysis

Clinical and biological characteristics, disease extent at the start of ^177^Lu-PSMA treatment, and prior treatments received are described for all patients included in this study, based on the nature of the variable studied, with the number of missing data presented where applicable. Quantitative or discrete data are summarized with median, 25^th^-75^th^ percentiles, minimum, maximum, mean and standard deviation. Categorical and binary data are described by the number and percentage of data points for each modality.

### Univariate analyses

To study the factors associated with early treatment discontinuation (primary endpoint), we distinguish between two groups of patients: those who were unable to receive more than 4 cycles and those who received 5 or 6 cycles of ^177^Lu-PSMA. We compare characteristics related to initial disease and prior treatments, clinical characteristics at the start of ^177^Lu-PSMA treatment and PET characteristics using ^68^Ga-PSMA before the initiation of ^177^Lu-PSMA treatment between these two groups using logistic regression models.

To investigate factors associated with progression or disease-related death, we used a semi-parametric Cox proportional hazards model to estimate the hazard ratios and associated *p*-values. In this model, time corresponds to the treatment regimen (C1 to C6). The event of interest is disease progression or death related to cancer. Patients who complete all 6 cycles without progression are censored at C6. Patients who discontinue treatment before C6 without progression and without disease-related death are censored at the date of their last received cycle. Risk estimation and its 95% confidence interval are performed using the Kaplan–Meier method.

### Multivariate analyses

All baseline variables are analyzed univariately first, and variables associated with survival with a *p*-value <0.2 are eligible for inclusion in the multivariate model. In cases of high correlation between two variables, the one most significantly associated with survival is preferred. Essential clinical characteristics for interpreting the multivariate analysis are also included in the model, regardless of their univariate significance.

For factors related to receiving 4 or fewer cycles, logistic regression models are employed. For factors associated with progression or disease-related death, Cox proportional hazards models are used.

### Repeated PSA measurements

Characteristics of patients who experience a reduction in PSA levels of more than 50% after the start of treatment (compared to before treatment) are compared using logistic regression models. Individual PSA trajectories are graphically represented.

Analyses are conducted under the responsibility of the Clinical Research Department of ICO, Promotion Unit, using appropriate statistical software (R or SAS). No imputation of missing data is planned in the protocol. A *p*-value <0.05 is considered statistically significant. In this exploratory study, no correction for alpha inflation is planned.

## Results

In this retrospective study, 63 patients were enrolled between February 2022 and December 2023. The average age at initial diagnosis was 62.2 ± 8.2 years and the average age at C1 was 71.8 ± 8.0 years.

At the time of diagnosis, 37 patients (58.7%) had metastatic disease, 16 patients (25.4%) had locally advanced disease and 10 patients (15.9%) had localized disease. A total of 34 patients (54%) had an ISUP (International Society of Urological Pathology) score of 4 or higher while 29 patients (46%) had an ISUP score below 4.

Before starting treatment, 56 patients (88.9%) had bone metastases, 35 patients (55.6%) had lymph node involvement, and 3 patients (7.9%) had visceral metastases (affecting the liver, lungs or brain). Of the group, 35 patients (55.6%) had received one line of NHA, 26 patients (41.3%) had received two lines, and 2 patients (3.2%) had received three lines. Additionally, 26 patients (41.3%) had undergone one line of taxane chemotherapy (Docetaxel only) while 37 patients (58.7%) had received two lines (Docetaxel followed by Cabazitaxel). The median initial PSA level was 67.7 ng/mL (95% CI: [19.8; 257.7]) and the median PSA doubling time before C1 was 2.2 months (95% CI: [1.4; 3.8]). On ^68^Ga-PSMA PET/CT scans, the median SUL_max_ was 23.7 (95% CI: [18.5; 53.1]) and the median TTV was 82.2 mL (95% CI: [18.0; 251.8]). Among these patients, 18 (28.6%) had a high PSG score, 26 (41.3%) had an intermediate PSG score and 19 (30.2%) had a low PSG score.

The average time from initial diagnosis to the start of the first treatment cycle was 8.3 ± 5.8 years. The average time between the ^68^Ga-PSMA PET/CT scan and the first treatment cycle was 1 ± 0.6 months. Resumed characteristics of the study population are presented in Table 2 and Appendix.

**Table 2:** Resumed characteristics of the study population.

Variable	Modality	4 cycles or less (*n* = 32 patients)	5 or 6 cycles (*n* = 31 patients)
Age at initial diagnosis	Min/Max	54.0/77.0	48.0/77.0
	Mean (std)	64.9 (5.8)	62.2 (8.2)
Age at first cycle of ^177^Lu-PSMA	Min/Max	60.0/91.0	52.0/87.0
	Mean (std)	72.6 (7.5)	71.0 (8.6)
ISUP score	< 4	17 (53.1%)	12 (38.7%)
	≥ 4	15 (46.9%)	19 (61.3%)
Extent at diagnosis	Localized or locally advanced	14 (43.8%)	12 (38.7%)
	Metastatic	18 (56.2%)	19 (61.3%)
Number of previous NHA	1	16 (50.0%)	19 (61.3%)
	2	16 (50.0%)	10 (32.3%)
	3	0 (0%)	2 (6.5%)
Number of previous taxane	1	11 (34.4%)	15 (48.4%)
	2	21 (65.6%)	16 (51.6%)
Baseline PSA level	Min/Max	0.2/1032.4	0.01/1066.0
	Mean (std)	198.3 (240.3)	149.9 (242.9)
PSA doubling time (month)	≥ 2	14 (43.8%)	22 (73.3%)
	< 2	18 (56.2%)	8 (26.7%)
	NA	0	1
Time between initial diagnosis and first cycle of ^177^Lu-PSMA	Min/Max	1.0/19.0	2.0/23.0
	Mean (std)	7.7 (5.6)	8.8 (6.0)
ECOG Performance Status	0	6 (18.8%)	11 (35.5%)
	≥ 1	26 (81.2%)	20 (64.5%)
Hb (g/dL)	≥ 10	21 (65.6%)	28 (90.3%)
	< 10	11 (34.4%)	3 (9.7%)
PLT (G/L)	> 150	29 (93.5%)	27 (87.1%)
	≤ 150	2 (6.5%)	4 (12.9%)
	NA	1	0
Albumin (g/L)	≥ 35	17 (56.7%)	26 (86.7%)
	< 35	13 (43.3%)	4 (13.3%)
	NA	2	1
Bone involvement	No	2 (6.2%)	5 (16.1%)
	Yes	30 (93.8%)	26 (83.9%)
Lymph node involvement	No	15 (46.9%)	13 (41.9%)
	Yes	17 (53.1%)	18 (58.1%)
Visceral metastases (liver, lung, brain)	No	25 (78.1%)	28 (90.3%)
	Yes	7 (21.9%)	3 (9.7%)

Min, Minimum; Max, Maximum; std, Standard Deviation; NA, Not Applicable; ^177^Lu, lutetium-117; NHA, Novel Hormonal Agent; PSMA, prostate-specific membrane antigen; ISUP, International Society of Urological Pathology; ECOG, Eastern Cooperative Oncology Group; PSA: prostate-specific antigen; Hb, hemoglobin; PLT, Platelets; SUL_max_, Maximum Standardized Uptake value – Lean body mass.

By the end of the study period, patients had received a median of 4 cycles of ^177^Lu-PSMA therapy. Of these, 32 patients (50.7%) received between 1 and 4 cycles and 31 patients (49.2%) received 5 or 6 cycles. Among those who received one to four cycles, 26 discontinued treatment due to disease progression (objectively confirmed by imaging: CT or bone scan), while 6 stopped for other reasons: 3 due to hematotoxicity, 1 due to death related to heart failure, 1 due to severe hyponatremia and 1 patient who opted to discontinue despite a favorable therapeutic response.

Among patients who received five or six cycles, 4 (6.3%) experienced disease progression (objectively confirmed by imaging) and 4 were awaiting their C6. Overall, 38 patients (60.3%) saw a reduction in PSA levels of 50% or more during treatment.

### Factors associated with early treatment discontinuation

In univariate analysis, the factors associated with early treatment discontinuation (four cycles or fewer) were as follows: a BMI < 25 vs. ≥ 25 kg/m^2^ (OR: 3.85; 95% CI [1.30–11.11]; *p* = 0.0148), a PSA doubling time < 2 months vs. ≥ 2 months (OR: 3.57; 95% CI [1.22–10.00]; *p* = 0.0206), hemoglobin <10 g/dL vs. ≥ 10 g/dL (OR: 4.89; 95% CI [1.21–19.75]; *p* = 0.0259), albumin <35 g/L vs. ≥ 35 g/L (OR: 4.97; 95% CI [1.39–17.82]; *p* = 0.0138), LDH > 250 IU/L vs. ≤ 250 IU/L (OR: 5.96; 95% CI [1.99–17.86]; *p* = 0.0014), and ALP > 125 IU/L vs. ≤ 125 IU/L (OR: 4.07; 95% CI [1.42–11.7]; *p* = 0.0091). On ^68^Ga-PSMA PET/CT, a lower SUL_max_ (per unit decrease: OR: 1.02; 95% CI [1.00–1.04]; *p* = 0.0361), a higher TTV compared to the median (OR: 4.62; 95% CI [1.60–13.35]; *p* = 0.0047) and a low vs. high PSG score (OR: 5.63; 95% CI [1.37–23.17]; *p* = 0.0166) were also associated with early treatment discontinuation. In multivariate analysis, BMI, PSA doubling time, PSG score, and TTV remained significantly associated with early treatment discontinuation.

The results of the statistical analyses are presented below in Tables 3, 4.

**Table 3:** Risk of early treatment discontinuation – univariate analysis.

Variable	Modality	OR	OR_inf_	OR_sup_	*p* value
Age at initial diagnosis	1 more unit	1.06	0.98	1.14	0.1387
Age at first cycle of ^177^Lu-PSMA	1 more unit	1.03	0.96	1.09	0.4281
ISUP score	≥ 4 vs. < 4	0.56	0.20	1.52	0.2528
Extent at diagnosis	Localized or locally advanced vs. metastatic	0.81	0.30	2.22	0.6847
	Locally advanced vs. localized	1.29	0.26	6.27	0.7560
	Metastatic vs. localized	0.95	0.23	3.83	0.9395
Initial treatment by radical prostatectomy	Yes vs. No	0.81	0.25	2.57	0.7144
Initial treatment by radiotherapy	Yes vs. No	0.73	0.27	1.98	0.5358
Enzalutamide	Yes vs. No	0.71	0.26	1.95	0.5032
Abiraterone	Yes vs. No	3.33	0.92	12.11	0.0674
Number of previous NHA	1 more unit	1.17	0.48	2.83	0.7316
Docetaxel	Yes vs. No	0.00	0.00	Inf	0.9915
Cabazitaxel	Yes vs. No	2.06	0.74	5.76	0.1672
Number of previous taxane based chemotherapy	1 more unit	1.79	0.65	4.93	0.2606
Baseline PSA	1 more unit	1.00	1.00	1.00	0.4278
PSA doubling time (month)	< 2 vs. ≥ 2	3.54	1.21	10.30	0.0206
Time between initial diagnosis and first cycle of ^177^Lu-PSMA (years)	1 more unit	0.97	0.89	1.06	0.4537
	> 6 vs. ≤ 6	0.83	0.31	2.22	0.7070
Regular need of level 2 or 3 analgesics	Yes vs. No	2.06	0.74	5.76	0.1672
ECOG Performance Status	≥ 1 vs. 0	2.38	0.75	7.55	0.1398
BMI (kg/m^2^)	≥ 25 vs. < 25	0.26	0.09	0.77	0.0148
Hb (g/dL)	< 10 vs. ≥ 10	4.89	1.21	19.75	0.0259
PLT (G/L)	≤ 150 vs. > 150	0.47	0.08	2.75	0.3989
WBC (G/L)	< 4 vs. ≥ 4	0.54	0.12	2.47	0.4259
Albumin (g/L)	< 35 vs. ≥ 35	4.97	1.39	17.82	0.0138
Corrected calcemia (mmol/L)	1 more unit	0.54	0.02	15.89	0.7180
LDH (U/L)	> 250 vs. ≤ 250	5.96	1.99	17.86	0.0014
ASAT (UI/L)	> 30 vs. ≤ 30	0.96	0.31	2.98	0.9414
ALAT (UI/L)	> 35 vs. ≤ 35	0.22	0.02	2.07	0.1844
gGT (UI/L)	> 45 vs. ≤ 45	1.47	0.51	4.21	0.4769
ALP (UI/L)	> 125 vs. ≤ 125	4.07	1.42	11.70	0.0091
SUL_max_	1 more unit	0.98	0.96	1.00	0.0361
	Higher vs. Lower	0.38	0.14	1.05	0.0615
PSG score	Intermediate vs. High	3.03	0.84	10.99	0.0912
	Low vs. High	5.63	1.37	23.17	0.0166
Total Tumor Volume	1 more unit	1.00	1.00	1.01	0.0475
	Higher vs. Lower	4.62	1.60	13.35	0.0047
Bone involvement	Yes vs. No	2.88	0.52	16.14	0.2279
	Single/oligometastatic vs. diffuse/widespread	0.56	0.17	1.91	0.3562
	No vs. diffuse/widespread	0.30	0.05	1.73	0.1775
Epiduritis	Yes vs. No	2.10	0.75	5.84	0.1553
Lymph node involvement	Yes vs. No	0.82	0.30	2.21	0.6934
Visceral metastases (liver, lung, brain)	Yes vs. No	2.61	0.61	11.21	0.1960
Time between ^68^Ga-PSMA PET/CT and first cycle of ^177^Lu-PSMA	1 more unit	1.23	0.50	3.06	0.6501

**Table 4:** Risk of early treatment discontinuation – multivariate analysis.

Variable	Modality	OR	OR_inf_	OR_sup_	Pr(>|t|)
Abiraterone	Yes vs. No	1.25	0.99	1.59	0.0703
PSA doubling time (month)	< 2 vs. ≥ 2	1.40	1.16	1.70	0.0013
BMI (kg/m^2^)	≥ 25 vs. < 25	0.74	0.61	0.90	0.0042
Albumin (g/L)	< 35 vs. ≥35	1.25	0.97	1.61	0.0980
LDH (U/L)	> 250 vs. ≤ 250	1.24	1.00	1.53	0.0559
ALAT (UI/L)	> 35 vs. ≤ 35	0.78	0.54	1.14	0.2065
PSG score	Intermediate vs. High	1.09	0.85	1.39	0.5007
	Low vs. High	1.43	1.07	1.90	0.0182
Total Tumor Volume	Higher vs. lower	1.31	1.02	1.67	0.0373
Visceral metastases (liver, lung, brain)	Yes vs. No	0.86	0.63	1.18	0.3617
Extent at diagnosis	M1 vs. M0	0.85	0.64	1.13	0.2726
Time between initial diagnosis and first cycle of ^177^Lu-PSMA (years)	> 6 vs. ≤ 6	0.84	0.63	1.12	0.2410

Additionally, aside from the PSG score, all these factors are significantly associated with disease progression and prostate cancer-specific mortality. This includes the presence of widespread bone involvement (HR: 2.34; 95% CI [1.14–4.78]; *p* = 0.0198) and pulmonary metastases (HR: 2.63; 95% CI [1.00–6.89]; *p* = 0.0493). In the multivariate analysis, BMI, PSA doubling time, albumin levels and TTV remain significant predictors. However, metastatic status at initial diagnosis (HR: 3.33; 95% CI [1.09–10.00]; *p* = 0.0357) and ECOG Performance Status ≥1 versus 0 (HR: 3.47; 95% CI [1.25–9.68]; *p* = 0.0173) also emerge as significant.

Detailed results are presented in the Appendix Tables 1, 2.

### Factors associated with poor PSA response

In univariate analysis, the clinico-biological factors associated with a reduction in PSA of less than 50% during treatment were as follows: BMI < 25 vs. ≥ 25 kg/m^2^ (OR: 3.85; 95% CI [1.30–11.11]; *p* = 0.0148), PSA doubling time < 2 vs. ≥ 2 months (OR: 3.54; 95% CI [1.21–10.30]; *p* = 0.0206), hemoglobin <10 g/dL vs. ≥ 10 g/dL (OR: 4.89; 95% CI [1.21–19.75]; *p* = 0.0259), albumin <35 g/L vs. ≥ 35 g/L (OR: 4.97; 95% CI [1.39–17.82]; *p* = 0.0138), LDH > 250 IU/L vs. ≤ 250 IU/L (OR: 5.96; 95% CI [1.99–17.86]; *p* = 0.0014), and ALP > 125 IU/L vs. ≤ 125 IU/L (OR: 4.07; 95% CI [1.42–11.7]; *p* = 0.0091). On ^68^Ga-PSMA PET/CT, a higher SUL_max_ (per unit increase: OR = 0.98; 95% CI [0.96–1.00]; *p* = 0.0361), a higher TTV compared to the median (OR: 4.62; 95% CI [1.60–13.35]; *p* = 0.0047) and a low vs. high PSG score (OR: 5.63; 95% CI [1.37–23.17]; *p* = 0.0166) were associated with a reduction in PSA of less than 50%.

In multivariate analysis, BMI, PSA doubling time, LDH levels, and PSG score remained significantly associated with PSA response. However, ECOG Performance Status ≥1 vs. 0 (HR: 1.34; 95% CI [1.05–1.71]; *p* = 0.0246) also became significant.

The PSA level trends and detailed statistical analysis results are presented in Appendix Figure 1 and Appendix Tables 3, 4.

### Overall survival from the first cycle of ^177^Lu-PSMA

**Figure 2:**
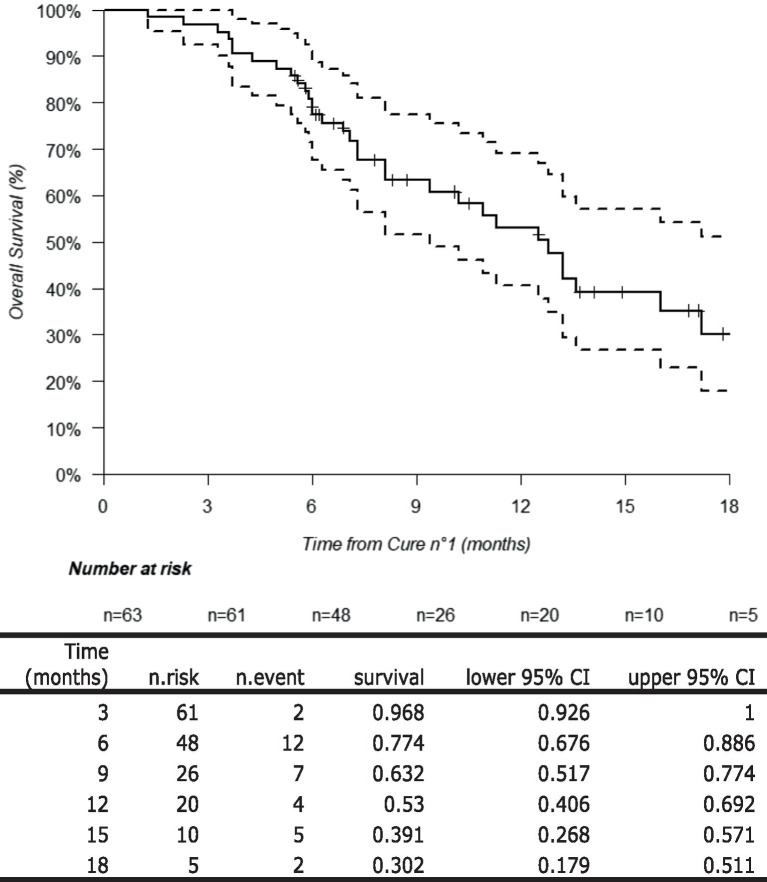
Overall survival of the study population.

The median follow-up period was 13.7 months (95% CI: 10.5–20.9). A total of 34 deaths were observed in the cohort with a median overall survival of 12.8 months (95% CI: 9.4–18.1). The overall survival of the cohort is depicted in Figure 2.

## Discussion

In this study, we assessed the impact of various clinico-biological factors and ^68^Ga-PSMA PET/CT findings on the response to ^177^Lu-PSMA treatment in patients with metastatic castration-resistant prostate cancer who had progressed after at least one line of taxane chemotherapy and NHA. We categorized the study population into two groups: patients who received 4 cycles or fewer of treatment versus those who received 5 to 6 cycles. We defined the group receiving 4 or fewer cycles as having a poor therapeutic response. This group also included 6 patients who discontinued treatment for reasons other than disease progression, allowing us to account for potential adverse effects of ^177^Lu-PSMA, comorbidities, and practical management issues.

### Impact of clinico-biological factors on response to ^177^Lu-PSMA

Our analysis identified several clinico-biological factors associated with an early treatment discontinuation, early progression and low PSA response rate: BMI < 25 kg/m^2^, short PSA doubling time, decreased hemoglobin and albumin level and increased LDH and ALP levels. While ECOG Performance Status ≥1 does not appear to be a predictor of early treatment discontinuation, it is associated with early progression and a poorer PSA response in multivariate analysis.

A low BMI, low serum albumin, and a high ECOG Performance Status generally reflect a state of malnutrition and are associated with a deterioration of the patient’s overall condition. Cancer-associated malnutrition is known to impair response to chemotherapy (18–20). Based on our findings, we suggest that malnutrition may be linked to a poorer response to ^177^Lu-PSMA. Further prospective studies are needed to confirm the impact of malnutrition on treatment outcomes with ^177^Lu-PSMA. Nonetheless, close monitoring of patient’s nutritional status before and during treatment is crucial. In cases of confirmed malnutrition, initiating a re-nutrition strategy could potentially improve therapeutic efficacy and survival.

Interestingly, higher BMI has been linked to better overall survival in cancer patients (21). This finding is paradoxical given that obesity is generally associated with increased all-cause mortality in the general population (22). This discrepancy might be due to one or more confounding factors, as BMI alone is not a reliable indicator of body composition or nutritional status. Body composition can vary widely by age and ethnicity, and individuals with the same BMI can have different distributions of lean and fat mass, which affects prognosis differently (23). For instance, a malnourished individual with sarcopenia but a normal BMI may have a worse prognosis compared to someone with a high BMI but without sarcopenia (24–26). Additionally, our study did not consider recent weight loss before treatment, which could be another confounding factor (27, 28).

While metastatic versus non-metastatic status at initial diagnosis is not a predictor of early treatment discontinuation, it is associated with early progression in multivariate analysis. In a retrospective study, Francini et al. demonstrated improved overall survival in patients with metachronous versus synchronous hormone-sensitive metastatic prostate cancer (29). Similarly, Bajeot et al. reported that synchronous metastatic patients were more symptomatic and had a higher metastatic burden compared to their metachronous counterparts (30). This leads us to hypothesize that the synchronous metastatic patients in our cohort likely had a more aggressive disease, which may have adversely affected their therapeutic response.

PSA doubling time is a dynamic measure of PSA progression and is widely recognized as one of the most reliable biological markers of prostate cancer aggressiveness. In a prospective study of 1,804 men with localized prostate cancer, D’Amico et al. showed that a PSA velocity exceeding 2 ng/mL per year in the year before diagnosis was associated with a tenfold higher risk of prostate cancer-specific mortality compared to those with a PSA velocity of 2 ng/mL per year or less, following radical prostatectomy (31). Likewise, in a retrospective study of 8,669 patients treated with surgery or radiotherapy, D’Amico et al. demonstrated that a PSA doubling time of less than 3 months was linked to significantly shorter prostate cancer-specific survival after biochemical recurrence, with a Hazard Ratio of 19.6 (32). Consistent with our findings, other studies have shown a significant link between PSA doubling time and progression-free survival after ^177^Lu-PSMA, with a short or negative doubling time predicting poor therapeutic response (33, 34). Based on our results and the existing literature, PSA doubling time also appears to reflect disease aggressiveness in the metastatic castration-resistant stage, with an impact on the response to ^177^Lu-PSMA. However, the initial PSA level before ^177^Lu-PSMA treatment does not appear to predict treatment response (35, 36).

In metastatic prostate cancer, elevated LDH levels are linked to shorter overall survival and poorer progression-free survival (PFS) (37). Similarly, in cases of bone metastatic prostate cancer, elevated ALP levels indicate increased osteoblastic activity and greater disease burden (38, 39), with a negative impact on overall survival (40). Other studies have confirmed that an elevation of these two markers is associated with a reduction in PFS after ^177^Lu-PSMA (34, 36, 41–45). In our cohort, elevation of these two biological markers also predict a poor therapeutic response to ^177^Lu-PSMA, as they are associated with earlier discontinuation of treatment. Thus, the elevation of these markers may also reflect aggressive metastatic disease.

A decrease in hemoglobin levels is frequently observed in cancer patients and is often due to multiple factors. These can include disease-related issues such as marrow infiltration, nutritional deficiencies, the inflammatory syndrome induced by the disease, or chemotherapy-induced toxicity affecting hematopoietic precursors. Low hemoglobin levels are known to be associated with reduced overall survival and lower treatment efficacy (46, 47). In our study, a hemoglobin level below 10 g/dL was strongly linked to early treatment discontinuation, early disease progression and a poorer PSA response with significant findings across all multivariate analyses. Therefore, investigating and addressing treatable causes of anemia may be beneficial for improving the response to ^177^Lu-PSMA.

Contrary to several previous reports, we did not find a significant association between regular use of analgesics of levels 2 or 3 and early treatment discontinuation, early progression or PSA response (36, 48).

Lastly, we could not demonstrate a significant association between previous treatments received at the metastatic castration-resistant stage and response to ^177^Lu-PSMA. However, the recent phase 3 clinical trial PSMAFore demonstrated that ^177^Lu-PSMA-617 treatment prolonged radiographic PFS versus androgen receptor pathway inhibitor change in taxane-naïve mCRPC patients (49). Moreover, another phase 2 clinical trial, UpFrontPSMA, demonstrated that a sequential treatment including ^177^Lu-PSMA followed by docetaxel resulted in a better therapeutic response based on PSA reduction compared to docetaxel alone (50). Finally, the TheraP clinical trial, which included 200 patients, found that ^177^Lu-PSMA was more effective than Cabazitaxel in reducing PSA levels in progressive mCRPC patients eligible for Cabazitaxel. This suggests that earlier use of ^177^Lu-PSMA before taxane chemotherapy failure may improve therapeutic response (51).

We were also unable to identify a significant link between initial disease characteristics at diagnosis (e.g., initial stage, histopathological grade), disease duration, type of local treatment (if any), and response to ^177^Lu-PSMA.

### Impact of metabolic factors on ^68^Ga-PSMA PET/CT

In our analysis of ^68^Ga-PSMA PET/CT, we identified that a low SUL_max_, a low PSG score, and a high TTV are associated with a poorer therapeutic response.

SUL_max_ measures the maximum activity within the most intense tumor voxel on ^68^Ga-PSMA PET/CT, adjusted for injected activity and the patient’s lean body mass. This unitless value reflects the tumor’s avidity for the tracer and thus the expression of membranous PSMA. Consequently, a lesion with high uptake of ^68^Ga-PSMA results in intense binding and a high SUL_max_. Other related measures include SUV_max_, which quantifies the activity of the most intense voxel relative to total body weight, and SUV_mean_, which measures the average activity within a segmented tumor volume, also relative to total body weight.

Our findings are consistent with those reported by Kuo et al. in their analysis of patients from the VISION study, which showed that high SUV_mean_ is associated with better PFS and overall survival (52). Similarly, Eisazadeh et al. found that a high SUL_max_ /Liver SUL_mean_ ratio was associated with longer PFS in patients treated with ^177^Lu-PSMA I&T (53). Additionally, a prospective study by Emmett et al. which included 14 patients, demonstrated that high SUV_max_ or SUV_mean_ values correlate with a better PSA response (54).

Our results also confirm the predictive value of the PSG score, as described by Hotta et al., which considers the overall intensity of PSMA expression across all tumor lesions. An uptake higher than that of the salivary glands for most lesions predicts a better response to ^177^Lu-PSMA. Conversely, an uptake lower than that of the salivary gland and, even more so, lower than that of the liver for most lesions predicts a poor response, with earlier treatment discontinuation and a less favorable PSA response (17). These results suggest that the intensity and homogeneity of lesions are key factors influencing the therapeutic response to ^177^Lu-PSMA.

Furthermore, a high TTV is a predictive factor for poor response, associated with early treatment discontinuation, early progression and a worse PSA response. This finding corroborates Kuo et al.’s results in their analysis of VISION data (55) and Wang et al.’s findings, which showed an association between high tumor volume and poorer PSA response (56). In line with this, we also observed that diffuse osteomedullary infiltration on ^68^Ga-PSMA PET/CT is associated with early progression.

In our study population, a low proportion of patients had undergone ^18^F − FDG PET/CT in addition to ^68^Ga-PSMA PET/CT before starting PLUVICTO^®^ to detect dedifferentiated tumor lesions. Consequently, we were unable to assess the impact of the presence of FDG-avid and PSMA-low lesions (FDG+/PSMA−) on therapeutic response. Rosar et al. demonstrated in their retrospective study that the FDG SUV_max_ /PSMA SUV_max_ ratio calculated across multiple target lesions per patient was predictive of therapeutic response after 2 cycles of ^177^Lu-PSMA. Specifically, an increase in this ratio (e.g., the presence of FDG+/PSMA− lesions) was associated with a poorer therapeutic response (57).

Several studies have demonstrated the adverse impact of visceral metastases, particularly hepatic involvement, on PFS and overall survival. A meta-analysis involving 1,504 patients indicated that the presence of visceral metastases is linked to both poorer biochemical response and shorter PFS and overall survival (58). We were unable to show this negative association in our cohort, likely due to limited statistical power: only 10 out of 63 patients had visceral involvement, and of these, only 3 received more than four cycles. However, we did find that pulmonary or pleural metastatic involvement was associated with early progression.

The observed overall survival in our study (median: 12.8 months) is lower than that reported in the VISION study (median: 15.3 months). This difference may be due to our more permissive inclusion criteria, which allowed us to include patients who were more severely impaired and had more extensive metastatic involvement. For instance, patients with a Superbonescan were excluded from the VISION study but were included in our cohort. Our study aimed to evaluate factors associated with the response to ^177^Lu-PSMA in real-world conditions, which naturally differ from those in a clinical trial setting. Moreover, a higher proportion of patients in our cohort received Cabazitaxel (60.3% compared to 41.8% in the VISION study). Indeed, according to the prospective TheraP trial, prior treatment with Cabazitaxel is associated with poorer PFS when followed by ^177^Lu-PSMA. Additionally, our study had a shorter median follow-up (13.7 months vs. 20.9 months), which could lead to an underestimation of overall survival. However, it is noteworthy that the proportion of patients achieving a PSA reduction of ≥50% during treatment was higher in our study compared to the VISION study (60.3% vs. 46.0%).

Additionally, it is interesting to note that the incidence of progression by C4 or earlier was higher among the initial patients treated in our cohort. This may be due to the overrepresentation of high tumor volumes and low PSG scores among these early patients. These patients also exhibited the most severe clinical and biological impairment. We can hypothesize that the latter patients were better selected, leading to more favorable therapeutic outcomes.

### Main limitations of the study

The primary limitations of this study include its retrospective design, which may introduce recall bias, the small sample size that limits statistical power, and its monocentric nature, which could introduce selection bias and center-specific effects. Specifically, this cohort consists of patients treated at a center specialized in cancer care (Centre de Lutte Contre le Cancer - CLCC) with management practices that might differ from those at other hospital settings, including conventional hospitals.

Additionally, we were unable to investigate the impact of molecular biomarkers on treatment response due to their limited use in routine practice. For example, androgen receptor gene amplification, determined through blood sampling, is associated with reduced PFS in several studies (59–61). Moreover, there are rare cases of patients with lesions showing high ^68^Ga-PSMA avidity and no FDG+/PSMA− lesions, who nevertheless exhibit a poor therapeutic response to ^177^Lu-PSMA. Some data suggest that genomic alterations such as BRCA2 mutation or neuroendocrine differentiation, could play a key role in treatment resistance (62).

A summarized comparison of our results with those of the previously mentioned studies is available in Appendix Table 5.

### Future perspectives

Currently, most studies focusing on predictive and prognostic factors associated with response to ^177^Lu-PSMA have a low level of evidence due to their retrospective design, often monocentric nature, small sample sizes, and highly heterogeneous methodologies. Conducting prospective studies to validate the predictive value of already identified factors would be beneficial. Such studies could facilitate the development of nomograms that could be used in clinical practice to more objectively select patients who are most likely to benefit from treatment. For instance, Gafita et al. developed nomograms to predict response to ^177^Lu-PSMA. However, this study is limited by its retrospective nature and the small size of the validation cohort (35).

Due to the high cost of treatment, its radiative nature, the complexity of its implementation, and potential adverse effects, patient selection is crucial.

This issue becomes even more significant considering that ^177^Lu-PSMA could potentially be used at earlier stages of prostate cancer in the future, significantly increasing the number of patients who would require treatment. This raises the important question of how to select between ^177^Lu-PSMA and other therapies based on the characteristics of the patients and their disease. Several clinical trials are currently investigating the efficacy of ^177^Lu-PSMA in earlier stages of prostate cancer. For instance, the PSMAfore trial which included 468 patients demonstrated that ^177^Lu-PSMA is superior to an androgen receptor pathway inhibitor change in taxane-naïve patients with mCRPC (63). Additionally, the ongoing PSMAddition trial is assessing the effectiveness of ^177^Lu-PSMA combined with standard of care (SoC) versus SoC alone in patients with metastatic hormone-sensitive prostate cancer (64).

Similarly, new radiopharmaceuticals using alpha-emitting isotopes, such as actinium-225 (^225^Ac − PSMA), have shown promising results in patients who have progressed after ^177^Lu-PSMA treatment (65).

## Conclusion

Our study identifies several predictive factors associated with a poor response to ^177^Lu-PSMA. Clinico-biologically, these factors include a body mass index (BMI) < 25 kg/m^2^, a PSA doubling time < 2 months, hemoglobin levels <10 g/dL, albumin levels <35 g/L, LDH > 250 IU/L and ALP > 125 IU/L. These factors are associated with a reduced likelihood of achieving a satisfactory therapeutic response and an increased risk of early treatment discontinuation.

On ^68^Ga-PSMA PET imaging, poor predictors include a low SUL_max_, high tumor volume (TTV) and a low PSG score.

The study’s findings underscore the importance of integrating clinico-biological parameters and advanced imaging results in the patient selection process for ^177^Lu-PSMA therapy. These predictive factors can aid in identifying patients who are less likely to benefit from the treatment, thereby optimizing patient management and potentially guiding the use of alternative or additional therapeutic strategies.

## Data Availability

The raw data supporting the conclusions of this article will be made available by the authors, without undue reservation.
