# Supplementary material for: Study of predictive factors for response to 177LU-PSMA in patients with metastatic castration-resistant prostate cancer
Source: Front Med (Lausanne). 2025 Mar 17;12:1538507. doi: 10.3389/fmed.2025.1538507 (PMC11955661; doi:10.3389/fmed.2025.1538507)
Supplement: Supplementary file 1 [file Data_Sheet_1.pdf]

APPENDIX

Table 1: Risk of early progression – Univariate analysis

Variable	Modality	HR	95 % CI inf	95 % CI sup	p.value
Age at initial diagnosis	1 more y	1.04	0.99	1.09	0.1642
Age at first cycle of ^{177}Lu-PSMA	1 more y	1.01	0.97	1.06	0.5142
ISUP score	≥ 4 vs < 4	0.74	0.37	1.50	0.4092
Extent at diagnosis	Localized or locally advanced vs metastatic	1.08	0.52	2.22	0.8381
	Locally advanced vs localized	1.26	0.38	4.18	0.7071
	Metastatic vs localized	1.25	0.42	3.67	0.6856
Initial treatment by radical prostatectomy	Yes vs No	0.68	0.28	1.65	0.3886
Initial treatment by radiotherapy	Yes vs No	0.92	0.45	1.88	0.8258
Enzalutamide	Yes vs No	0.85	0.42	1.75	0.6665
Abiraterone	Yes vs No	3.20	0.97	10.55	0.0555
Number of previous NHA	1 more line	1.22	0.68	2.19	0.4978
Docetaxel	Yes vs No	3,36	0.00	Inf	0.9971
Cabazitaxel	Yes vs No	1.85	0.85	4.03	0.1192
Number of previous taxane based chemotherapy	1 more line	1.90	0.87	4.13	0.1051
Baseline PSA level	1 more unit	1.00	1.00	1.00	0.1637
PSA doubling time (month)	< 2 vs ≥ 2	2.08	1.02	4.23	0.0441
Time between initial diagnosis and first cycle of ^{177}Lu-PSMA (years)	1 more y	0.97	0.91	1.04	0.3899
	> 6 vs ≤ 6	0.75	0.37	1.53	0.4293
Regular need of level 2 or 3 analgesics	Yes vs No	1.85	0.85	4.03	0.1192
ECOG Performance Status	≥ 1 vs 0	1.76	0.76	4.10	0.1875
BMI (kg/m^2)	≥ 25 vs < 25	0.34	0.17	0.69	0.0029
Hb (g/dL)	< 10 vs ≥ 10	3.27	1.55	6.87	0.0018
PLT (G/L)	≤ 150 vs > 150	0.69	0.16	2.91	0.6155
WBC (G/L)	< 4 vs ≥ 4	0.59	0.18	1.95	0.3872
Albumin (g/L)	< 35 vs ≥ 35	2.65	1.27	5.55	0.0095
Corrected calcemia (mmol/L)	1 more unit	0.76	0.06	9.83	0.8318
LDH (U/L)	> 250 vs ≤ 250	3.38	1.55	7.37	0.0022
ASAT (UI/L)	> 30 vs ≤ 30	1.24	0.57	2.69	0.5923
ALAT (UI/L)	> 35 vs ≤ 35	0.67	0.16	2.79	0.5776
gGT (UI/L)	> 45 vs ≤ 45	1.69	0.83	3.45	0.1500
ALP (UI/L)	> 125 vs ≤ 125	3.10	1.45	6.60	0.0034

Variable	Modality	HR	95 % CI inf	95 % CI sup	p.value
SUL_{max}	1 more unit	0.99	0.97	1.00	0.0891
	Higher vs lower	0.66	0.32	1.35	0.2576
PSG score	Intermediate vs High	2.40	0.87	6.63	0.0901
	Low vs High	2.68	0.93	7.75	0.0689
Total Tumor Volume (mL)	1 more unit	1.00	1.00	1.00	0.0066
	Higher vs lower	3.46	1.59	7.54	0.0018
Bone involvement	Yes vs No	2.20	0.52	9.24	0.2806
	Single/oligometastatic vs diffuse/widespread	0.53	0.20	1.38	0.1943
	No vs diffuse/widespread	0.39	0.09	1.67	0.2051
Epiduritis	Yes vs No	2.00	0.99	4.07	0.0545
Lymph node involvement	Yes vs No	0.83	0.41	1.69	0.6118
Pleural/lung metastases	Yes vs No	2.63	1.00	6.89	0.0493
Visceral metastases (liver, lung, brain)	Yes vs No	2.11	0.91	4.91	0.0829
Time between ^{68}Ga-PSMA PET/CT and first cycle of ^{177}Lu-PSMA	1 more month	1.22	0.70	2.13	0.4805

Table 2: Risk of early progression – Multivariate analysis

Variable	Modality	HR	HR_{inf}	HR_{sup}	p.value
Abiraterone	Yes vs No	4.14	0.90	19.12	0.0689
PSA doubling time (month)	< 2 vs ≥ 2	5.07	2.18	11.78	0.0002
Extent at diagnosis	M1 vs M0	0.30	0.10	0.92	0.0357
Time between initial diagnosis and first cycle of ^{177}Lu-PSMA (years)	> 6 vs ≤ 6	0.31	0.09	1.03	0.0564
ECOG Performance Status	≥ 1 vs 0	3.47	1.25	9.68	0.0173
BMI (kg/m^2)	≥ 25 vs < 25	0.18	0.07	0.45	0.0003
Albumin (g/L)	< 35 vs ≥ 35	3.13	1.13	8.67	0.0281
Total Tumor Volume (mL)	Higher vs lower	3.94	1.45	10.70	0.0071
Visceral metastases (liver, lung, brain)	Yes vs No	1.66	0.63	4.34	0.3028

Table 3: Risk of poor PSA response – Univariate analysis

Variable	Modality	OR	OR_{inf}	OR_{sup}	p.value
Age at initial diagnosis	1 more unit	1.03	0.96	1.11	0.3599
Age at first cycle of ^{177}Lu-PSMA	1 more unit	1.03	0.96	1.09	0.4281
ISUP score	≥ 4 vs < 4	0.56	0.20	1.52	0.2528
Extent at diagnosis	Localized or locally advanced vs metastatic	0.81	0.30	2.22	0.6847
	Locally advanced vs localized	1.29	0.26	6.27	0.7560
	Metastatic vs localized	0.95	0.23	3.83	0.9395
Initial treatment by radical prostatectomy	Yes vs No	0.81	0.25	2.57	0.7144

Variable	Modality	OR	OR_{inf}	OR_{sup}	p.value
Initial treatment by radiotherapy	Yes vs No	0.73	0.27	1.98	0.5358
Enzalutamide	Yes vs No	0.71	0.26	1.95	0.5032
Abiraterone	Yes vs No	3.33	0.92	12.11	0.0674
Number of previous NHA	1 more unit	1.17	0.48	2.83	0.7316
Docetaxel	Yes vs No	0.00	0.00	Inf	0.9915
Cabazitaxel	Yes vs No	2.06	0.74	5.76	0.1672
Number of previous taxane based chemotherapy	1 more unit	1.79	0.65	4.93	0.2606
Baseline PSA level	1 more unit	1.00	1.00	1.00	0.4278
PSA doubling time (month)	< 2 vs ≥ 2	3.54	1.21	10.30	0.0206
Time between initial diagnosis and first cycle of ^{177}Lu-PSMA (years)	1 more unit	0.97	0.89	1.06	0.4537
	> 6 vs ≤ 6	0.83	0.31	2.22	0.7070
Regular need of level 2 or 3 analgesics	Yes vs No	2.06	0.74	5.76	0.1672
ECOG Performance Status	≥ 1 vs 0	2.38	0.75	7.55	0.1398
BMI (kg/m2)	≥ 25 vs < 25	0.26	0.09	0.77	0.0148
Hb (g/dL)	< 10 vs ≥ 10	4.89	1.21	19.75	0.0259
PLT (G/L)	≤ 150 vs > 150	0.47	0.08	2.75	0.3989
WBC (G/L)	< 4 vs ≥ 4	0.54	0.12	2.47	0.4259
Albumin (g/L)	< 35 vs ≥ 35	4.97	1.39	17.82	0.0138
Corrected calcemia (mmol/L)	1 more unit	0.54	0.02	15.89	0.7180
LDH (U/L)	> 250 vs ≤ 250	5.96	1.99	17.86	0.0014
ASAT (UI/L)	> 30 vs ≤ 30	0.96	0.31	2.98	0.9414
ALAT (UI/L)	> 35 vs ≤ 35	0.22	0.02	2.07	0.1844
gGT (UI/L)	> 45 vs ≤ 45	1.47	0.51	4.21	0.4769
ALP (UI/L)	> 125 vs ≤ 125	4.07	1.42	11.70	0.0091
SUL_{max}	1 more unit	0.98	0.96	1.00	0.0361
	Higher vs Lower	0.38	0.14	1.05	0.0615
PSG score	Intermediate vs High	3.03	0.84	10.99	0.0912
	Low vs High	5.63	1.37	23.17	0.0166
Total Tumor Volume (mL)	1 more unit	1.00	1.00	1.01	0.0475
	Higher vs Lower	4.62	1.60	13.35	0.0047
Epiduritis	Yes vs No	2.10	0.75	5.84	0.1553
Bone involvement	Yes vs No	2.88	0.52	16.14	0.2279
	Single/oligometastatic vs diffuse/widespread	0.56	0.17	1.91	0.3562
	No vs diffuse/widespread	0.30	0.05	1.73	0.1775
Lymph node involvement	Yes vs No	0.82	0.30	2.21	0.6934
Visceral metastases (liver, lung, brain)	Yes vs No	2.61	0.61	11.21	0.1960
Time between ^{68}Ga-PSMA PET/CT and first cycle of ^{177}Lu-PSMA	1 more unit	1.23	0.50	3.06	0.6501

Table 4: Risk of poor PSA response – Multivariate analysis

Variable	Modality	OR	OR_{inf}	OR_{sup}	p.value
PSA doubling time (month)	< 2 vs ≥ 2	1.34	1.09	1.67	0.0092
ECOG Performance Status	≥ 1 vs 0	1.34	1.05	1.71	0.0246
BMI (kg/m^2)	≥ 25 vs < 25	0.79	0.63	0.97	0.0328
LDH (U/L)	> 250 vs ≤ 250	1.28	1.02	1.60	0.0407

Variable	Modality	OR	OR_{inf}	OR_{sup}	p.value
PSG score	Intermediate vs High	1.11	0.86	1.43	0.4262
	Low vs High	1.42	1.04	1.94	0.0320
Visceral metastases (liver, lung, brain)	Yes vs No	0.75	0.46	1.25	0.2751
Pleural/Lung metastases	Yes vs No	1.60	0.88	2.91	0.1341
Extent at diagnosis	M1 vs M0	0.98	0.72	1.32	0.8694
Time between initial diagnosis and first cycle of ^{177}Lu-PSMA (years)	> 6 vs ≤ 6	1.02	0.77	1.36	0.8816

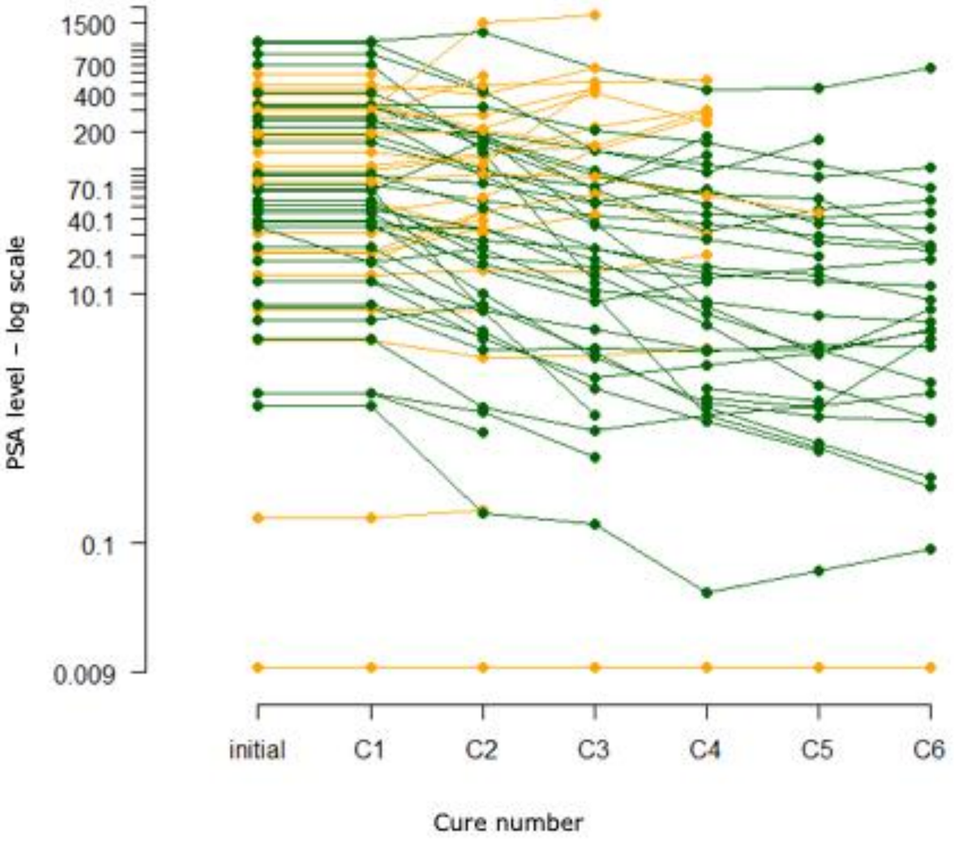

Figure 1: PSA level trends for each patient

Table 5: Comparison of our study results with key studies on predictive factors for 177Lu-PSMA response

	Short PSA doubling time	Initial PSA level	Elevated LDH or ALP levels	Low hemoglobin levels	Regular need of analgesics of levels 2/3	Previous mCRPC treatment received	Low SUV_{mean} Low SUV_{max} Low SUL_{max}	Low PSG-score	High TTV	High FDG avidity/low PSMA expression	Visceral metastases	Androgen receptor gene amplification
Our study	Predictive	Non-significant	Predictive	Predictive	Non-significant	Non-significant	Predictive	Predictive	Predictive	Not evaluated	Non-significant	Not evaluated
Suman S. (33)	Predictive											
Telli T. (34)	Predictive		Predictive	Predictive						Predictive	Predictive	
Gafita A. (35)				Predictive		Predictive	Predictive				Predictive	
Ferdinandus J. (36)		Non-significant	Predictive	Predictive	Predictive	Non-significant	Non-significant				Non-significant	
Rasul S. (16)		Non-significant	Non-significant	Predictive		Non-significant					Non-significant	
Barber TW. (41)		Non-significant	Predictive	Predictive							Non-significant	
Heck MM. (42)		Non-significant	Predictive	Non-significant		Non-significant					Predictive	
Rathke H. (43)		Predictive	Predictive				Predictive					
Bräuer A. (44)			Predictive									
Rahbar K. (45)			Predictive			Non-significant					Predictive	
Ahmadzadehfar H. (48)		Non-significant			Predictive	Non-significant						
Morris MJ. (49)						Predictive						
Azad AA. (50)						Predictive						
Hofman MS. (51)						Predictive						
Kuo P. (52)							Predictive		Predictive			
Eisazadeh R. (53)		Non-significant					Predictive					
Emmet L. (54)							Predictive		Non-significant	Predictive	Non-significant	
Hotta M. (17)								Predictive				
Kuo P. (55)							Predictive		Predictive			
Wang G. (56)			Predictive						Predictive			
Rosar F. (57)							Predictive			Predictive		
Satapathy S. (58)											Predictive	
Vanwelkenhuyzen J. (59)												Predictive
De Giorgi U. (60)												Predictive
Sun M. (61)												Predictive

For each study, predictive factors of response to 177Lu-PSMA are highlighted in green, non-significant factors are highlighted in red, and empty cells correspond to factors that were not evaluated.
